# Analysis of CpG methylation sites and CGI among human papillomavirus DNA genomes

**DOI:** 10.1186/1471-2164-12-580

**Published:** 2011-11-25

**Authors:** Silvia C Galván, Martha Martínez-Salazar, Víctor M Galván, Rocío Méndez, Gibran T Díaz-Contreras, Moisés Alvarado-Hermida, Rogelio Alcántara-Silva, Alejandro García-Carrancá

**Affiliations:** 1 Instituto de Investigaciones Biomédicas, Universidad Nacional Autónoma de México, México; 2 Departamento El Hombre y su Ambiente, Universidad Autónoma Metropolitana-Xochimilco, México; 3 Instituto Nacional de Cancerología, Secretaría de Salud, México; 4 Facultad de Ingeniería, Universidad Nacional Autónoma de México, México

## Abstract

**Background:**

The Human Papillomavirus (HPV) genome is divided into early and late coding sequences, including 8 open reading frames (ORFs) and a regulatory region (LCR). Viral gene expression may be regulated through epigenetic mechanisms, including cytosine methylation at CpG dinucleotides. We have analyzed the distribution of CpG sites and CpG islands/clusters (CGI) among 92 different HPV genomes grouped in function of their preferential tropism: cutaneous or mucosal. We calculated the proportion of CpG sites (PCS) for each ORF and calculated the expected CpG values for each viral type.

**Results:**

CpGs are underrepresented in viral genomes. We found a positive correlation between CpG observed and expected values, with mucosal high-risk (HR) virus types showing the smallest O/E ratios. The ranges of the PCS were similar for most genomic regions except *E4*, where the majority of CpGs are found within islands/clusters. At least one CGI belongs to each *E2/E4 *region. We found positive correlations between PCS for each viral ORF when compared with the others, except for the LCR against four ORFs and *E6 *against three other ORFs. The distribution of CpG islands/clusters among HPV groups is heterogeneous and mucosal HR-HPV types exhibit both lower number and shorter island sizes compared to cutaneous and mucosal Low-risk (LR) HPVs (all of them significantly different).

**Conclusions:**

There is a difference between viral and cellular CpG underrepresentation. There are significant correlations between complete genome PCS and a lack of correlations between several genomic region pairs, especially those involving LCR and *E6*. *L2 *and *L1 *ORF behavior is opposite to that of oncogenes *E6 *and *E7*. The first pair possesses relatively low numbers of CpG sites clustered in CGIs while the oncogenes possess a relatively high number of CpG sites not associated to CGIs. In all HPVs, *E2/E4 *is the only region with at least one CGI and shows a higher content of CpG sites in every HPV type with an identified *E4*. The mucosal HR-HPVs show either the shortest CGI size, followed by the mucosal LR-HPVs and lastly by the cutaneous viral subgroup, and a trend to the lowest CGI number, followed by the cutaneous viral subgroup and lastly by the mucosal LR-HPVs.

## Background

Human papillomavirus (HPV) constitute a group of over 100 different types. HPV infect stratified epithelia, both mucosal and cutaneous and associate with benign and malignant proliferative disorders. Viral types that preferentially infect mucosal epithelia are grouped into either a low-risk group (LR-HPV) not associated with cancer, or into a high-risk group (HR-HPV) whose members are found in almost all cases of cervical cancer [1]. HPV are non-lytic, non-enveloped, icosahedral-shaped viruses with a circular, double-stranded DNA genome of approximately 8.0 kb that is functionally divided into two coding regions (denoted *E *for early or *L *for late) and one regulatory region or LCR. The *E *region includes six major open-reading frames (ORFs) encoding functional proteins (E1, E2 and E4) and oncoproteins (E5, E6 and E7), and the *L *region encodes the two capsid proteins, L1 and L2. *E4 *is expressed in both early and late stages of the viral life cycle [2].

HPV gene expression is mainly regulated at the transcriptional and post-transcriptional levels and several studies have suggested that viral DNA methylation may be associated with viral gene expression and cancer progression [3-6].

Most of the work on HPV methylation has been carried out on the two main viral types involved in cervical cancer (types 16 and 18). In both genomes there is a progressive increase in methylation from asymptomatic carriers, through benign lesions and pre-malignant disease, to cancer tumors. Nevertheless, there is heterogeneity of CpG methylation in viral genomes derived from clinical specimens [4-7], and the exact role of viral DNA methylation remains unclear.

Furthermore, based on Epstein-Barr virus studies it has been proposed that viral genome methylation may enable a proportion of infected cells to survive cytotoxic T-cell immune surveillance [8,9], and studies on adenovirus late viral genes suggest that they are more sensitive to methylation than early ones [10]. In the case of HPV *L2 *and *L1 *genes, they have been proposed to be preferentially recognized by the cellular methylation machinery [11,12].

Methylation is the only known covalent modification of DNA in eukaryotes and plays an important regulatory role in vertebrates by silencing specific genes during development and cell differentiation. Cytosine methylation occurs at the 5'-position of the pyrimidine ring, mainly within a CpG context (m5CpG), although methylation of cytosines in different contexts has recently been described [13,14] and 5-hydroxymethylcytosine (5 hmC), a novel DNA modification was reported [15].

Usually, the presence of m5CpG in genomic DNA is associated with chromatin condensation and inactivation of gene expression. Nevertheless, it is not clear whether the primary evolutionary role of DNA methylation is transcriptional silencing or a host defense system against endogenous or exogenous parasitic sequence elements [16].

In the genomes of higher eukaryotes, CpG dinucleotides are usually underrepresented, from one third down to 5% of their expected frequency [17-20]. The mechanism proposed to explain this underrepresentation, first recognized in prokaryotic systems [21], is that CpG sites are mutagenic due to the frequent conversion of methylcytosines to thymines through deamination [22].

Similar to their hosts, CpG dinucleotides are underrepresented in the majority of small DNA viruses, although to a lesser extent [23]. It has been proposed that low CpG frequencies may either allow viruses to avoid methylation by host methyltransferases and thus maximize their transcriptional efficiency, or could be a means of reducing CpG mediated immune responses [24].

The general view is that CpG sites in vertebrates, grouped within clusters or islands (CGI), are mostly unmethylated in promoter regions of transcriptionally active genes, and methylated in promoter regions of transcriptionally inactive genes. In contrast, methylation in body genes seems to be evolutionarily conserved and plays an important role in tissue- or cell-specific alternative promoter regulation. Furthermore, tumor cells usually show hypomethylation of the majority of the genome and methylation of CGIs in promoter regions of tumor suppressor genes [14,25].

According to Gardiner-Garden and Frommer [26] a CGI is defined as a DNA fragment of at least 200 bp containing at least 50% of CpG's, with a CpG Observed/Expected (O/E) ratio of 0.60 or higher and at least a 100 bp gap in between different CGIs. This definition, despite its popularity, has raised many criticisms and various different proposals have been postulated [27-31]. Han et al., [32] recently compared three computational algorithms for CpG island identification and, based on vertebrate gene function and other genomic factors, the Takai and Jones criteria performance was the best. The analysis of 132 CGIs across the entire human chromosome 21 allowed researchers to identify several attributes associated with either methylation-resistance or methylation-sensitivity of CGIs [33].

The majority of the proposals for CGI identification and criteria to analyze methylation predisposition at CpG islands cannot be directly applied to HPV genomes because of their small size. In addition, lack of multiple promoter regions and the scarce data on methylation of different viral types make this scenario difficult.

Because in our view, understanding DNA methylation may benefit from our knowledge of CpG distribution along different viral genomes, we have analyzed genomic and regional proportions of CpG sites (PCS) and their arrangement into CGIs, in most human papillomavirus types sequenced to date. To our knowledge, there has been no comprehensive analysis of CGIs in virus genomes. We propose hypotheses about their functional roles, considering HPVs as both a single group or as subgroups in function of their preferential tropism (cutaneous and mucosal) and their associated risk to induce cancer (mucosal low- and high-risk).

## Results

### CpG sites are underrepresented among HPV genomes, although to a lesser extent than in their hosts

After identifying all CpG sites among 92 available HPV DNA sequences, we calculated the expected CpG value for each viral genome. The expected number of CpG sites was calculated as the number of 'C's multiplied by the number of 'G's in the viral genome, divided by the genome size.

**Figure 1:**
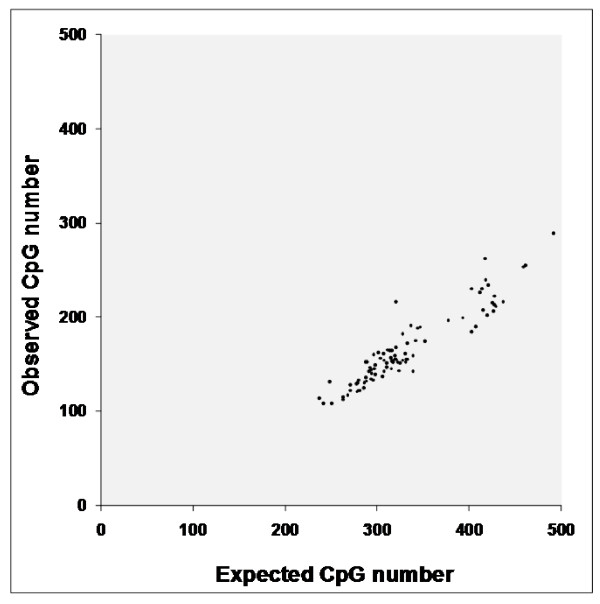
**Relationship between CpG observed and expected values for HPV genomes**.

When we compared the CpG observed vs. the expected values for each viral type, a significant difference was clear (one side p = 0, Chi square test). In addition, a positive correlation was found (two sided p = 4.30E-37, rho Spearman Rank Correlation; Figure 1) indicating a similar proportion of CpGs diminishing irrespective of viral type. As anticipated, CpG sites are underrepresented in viral genomes, although to a lesser extent than in their human host genome [17,20]. The average difference between means is 50.5% (ranging from 32.5% to 58.1%).

Interestingly, most HR-HPV infecting mucosal epithelia show CpG O/E ratios below the O/E average ratio (0.50); only five HR-HPV types (18, 26, 45, 39, and 68) show differences slightly above the average ratio (additional file 1, Table S1). In fact, the HR-HPVs possess the lowest average (0.47) CpG O/E ratio which means that this viral sub-group shows a deeper CpG underrepresentation. CpG O/E average ratios for LR-and cutaneous HPV types are 0.49 and 0.51, respectively.

In Figure 1, we can observe CpG values as a function of the expected values for all 92 sequenced HPV genomes. The calculated O/E ratio for each individual nucleotide among different viral genomes indicates that C (0.8) and G (0.9) are underrepresented while A (1.2) and T (1.1) are overrepresented within HPV genomes.

### HPV *E4 *is the region with the highest proportion of CpGs

**Figure 2:**
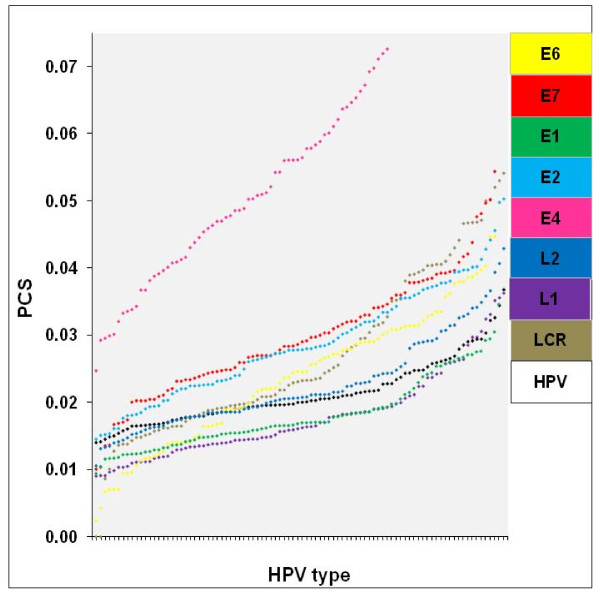
**Distribution of HPV types in function of regional PCS values**. Every data set is graphed according to the order of each particular regional PCS, from the lowest to the maximum. PCS is the number of CpG sites divided by the total number of nucleotides in the regional sequence.

We calculated each PCS as the number of CpG sites divided by the total number of nucleotides in the considered sequence. Then we plotted PCS for each viral region in ascending order (Figure 2) independently of the viral type. This means that each x axis value may represent more than one viral type.

From Figure 2 (and additional file 2, Table S2, where all analyzed viral genomes are listed), it is clear the *E4 *ORF possesses the highest regional PCS value (61 out of 68 HPV types where this region has been identified; see Methods section for HPV types lacking reported ORFs), and that the *E2 *region (where *E4 *is co-located) does not show similar PCS values (average *E2 *PCS without HPV types lacking *E4 *was 0.028). In three out of 68 viral types, *E7 *exhibits the highest PCS values while the LCR region shows the highest PCS values in four out of 68 types.

Regional average PCS (and range) for individual ORFs and the LCR are as follows: *E6*, 0.024 (0.002 to 0.045); *E7*, 0.030 (0.010 to 0.054); *E1*, 0.018 (0.009 to 0.035); *E2*, 0.029 (0.014 to 0.050); *E4*, 0.051 (0.025 to 0.088); *L2*, 0.023 (0.011 to 0.043); *L1*, 0.018 (0.009 to 0.036); and LCR, 0.027 (0.009 to 0.054).

When HPVs are grouped in function of their preferential tropism, the *E4 *ORF PCS averages are the highest when compared to any other region. In fact, *E4 *ORF PCS averages are quite close, irrespective of type: for cutaneous, 0.054 and for mucosal, 0.049, similar to when mucosal HPVs were grouped in function of risk: low risk, 0.050 and high risk, 0.048. Furthermore, *E4 *average PCS values are around twice the highest average PCS from any other region in each viral group/subgroup (data not shown).

Interestingly, O/E ratios for A (1.16) and G (0.96) individual nucleotides in the *E4 *ORF, follow the same trend found in the entire viral genome, however, T (0.68) seems underrepresented and C (1.20) overrepresented, compared to whole genome values. When Arc-sin transformed O/E ratios for single nucleotides in *E4 *and in the whole genome were compared, we found that the two sets of data are significantly different (p = 4.94E-14; t test), even though they are significantly correlated (p = 006; Pearson Product Moment Correlation). PCS were Arc-sin transformed in order to properly use statistical procedures, as mentioned in the Methods section.

### HPV type distributions in function of regional proportion of CpG sites are dissimilar

HPV types were ordered in function of regional Arc-sin transformed PCS (AsPCS) and compared to each other using a Pearson linear correlation coefficient and two sided t tests. Results are shown in Table 1, where it can be seen that 36 out of 45 are significant correlations. This means that any viral type closely maintains its relative position in function of each significant correlated couple of regional PCS. There is a lack of correlation between LCR PCS and those from the *E1, E4, L2 *and *L1 *regions, as well as the average of the ORFs; between *E6 *PCS and those from the *E7, E2 *and *E4 *regions; and between *E7 *PCS and that from the *L2 *region.

**Table 1:** p values for correlations* between regional AsPCS values

Genomic Regions	All ORFs	*E6*	*E7*	*E1*	*E2*	*E4*	*L2*	*L1*	LCR
**HPV genome**	0.0000	0.0006	0.0000	0.0000	0.0000	0.0000	0.0000	0.0000	0.0016
**All ORFs**		0.0000	0.0000	0.0000	0.0000	0.0000	0.0000	0.0000	0.2493
***E6***			0.9080	0.0000	0.2674	0.2453	0.0000	0.0000	0.0000
***E7***				0.0112	0.0015	0.0293	0.0965	0.0019	0.0286
***E1***					0.0000	0.0001	0.0000	0.0000	0.9719
***E2***						0.0000	0.0000	0.0059	0.0000
***E4***							0.0036	0.0003	0.0564
***L2***								0.0000	0.7086
***L1***									0.1081

*Pearson Product Moment Correlation and two sided t test.

### The distribution of CpG islands/clusters among HPVs is heterogeneous

CGI location is summarized in Table 2 (and described in additional file 3, Table S3). There are in average 3 CGIs per HPV genome (ranging from 1 to 8) and the average island size is 394 bp, ranging from 203 to 1379 bp. Twenty two HPV types possess a single CGI with a average size of 447 bp (ranging from 210 to 835); 25 types possess two CGIs with a average size of 360 bp (ranging from 215 to 562); 15 types possess three CGIs with a average size of 303 bp (ranging from 234 to 426); 10 types possess four CGIs, average size of 365 bp (ranging from 241 to 510); 6 types possess five CGIs, average size of 481 bp (ranging from 265 to 724); 6 types possess six CGIs, average size of 477 bp (ranging from 391 to 575); 7 types possess seven CGIs, average size of 381 bp (ranging from 344 to 424), and just one type (HPV 57) possesses eight CGIs with an average size of 505 bp (ranging from 209 to 1159). None of the viruses lack CGIs, and in all cases at least one CGI belongs to the *E2/E4 *region.

**Table 2:** Proportion of CpG islands per HPV for each viral group

CGIs	*E6*	*E7*	*E1*	*E2*	*E4*	*L2*	*L1*	LCR	HPV Genome
**High-Risk HPVs**	0.07	0.47	0.33	1.00	1.00	0.13	0.00	0.00	1.60
**Low-Risk HPVs**	0.32	0.55	1.14	1.03	1.00	1.00	0.55	0.46	3.93

**Mucosal HPVs**	0.22	0.51	0.83	1.02	1.00	0.66	0.34	0.28	3.04
**Cutaneous HPVs**	0.16	0.49	0.84	1.00	1.00	0.58	0.44	0.39	3.02

**All HPVs**	0.19	0.50	0.84	1.01	1.00	0.62	0.39	0.33	3.03

**Maximum number**	1	1	2	2	1	3	4	2	8
**Minimum number**	0	0	0	1	1	0	0	0	1

### HPV *E2/E4 *is the region with the highest number of CGIs

We found that the viral region with the highest number of CGIs (CGIn) is *E2/E4*; each viral type possessing at least one CGI in this region. It is important to make clear that in the case of *E4*, as in any other region, we have only taken into account those HPV types where this ORF has been identified. Other regions which most commonly contain CGIs include the *E1, E7 *and *L2 *ORFs which have CGIs in at least half of HPV types; the *L1 *ORF and LCR regions with one CGI in 25 to 50% of viral types, and finally, the region where less than 25% of the viral types possess one CGI is *E6*.

In most types, there is not an exact correspondence between whole genome CGIn and regional CGIn because when an island/cluster is distributed in more than one viral region, it is taken into account in every comprehended region; even if there is just one CGI in the whole genome. This applies particularly to the *E2 *and *E4 *regions because the *E4 *ORF is located within the *E2 *ORF, although in a different reading frame (see Table 2).

### High-risk HPV types exhibit lesser and shorter CpG islands/clusters

When grouped by tropism, CGI average sizes and ranges are as follows: cutaneous HPV types averaging 446 bp (from 203 to 1211 bp); all mucosal HPV types, 345 bp (from 205 to 1379 bp); mucosal LR-HPV types, 363 bp (from 205 to 1379 bp), and mucosal HR-HPV types, 275 bp (from 210 to 410 bp). Notably, when CGI frequencies per size were compared, we found significant differences between cutaneous and mucosal HPVs (p < 0.0005) as well as between mucosal LR- and HR-HPVs (p < 0.0001, Chi square test). Additionally, both cutaneous and mucosal viral types share an average of 3.0 CGIs per viral genome (ranging from 1 to 8), and among mucosal types, LR-HPVs show an average of 3.93 CGIs per viral genome in contrast to HR-HPVs that show only 1.60 CGIs. When compared frequencies per number of CGI, we also found significant differences between cutaneous and mucosal HPVs (p < 0.005) as well as between mucosal LR- and HR-HPVs (p < 0.001, Chi square test).

Thirty out of 45 cutaneous and 32 out of 47 mucosal types possess 3 or less CGIs, and 15 out of 45 cutaneous and 15 out of 47 mucosal types possess more than 3 CGIs. In the mucosal group, 14 out of 29 LR- and all 18 HR-HPVs possess 3 or less CGIs, and 15 out of 29 LR-HPVs posses more than 3 CGIs (additional file 3, Table S3).

Although CGIs seem regionally distributed in a similar manner between cutaneous and mucosal types (except for *E6, L1 *and LCR), there is a notable difference between the proportion of CGIs among both mucosal HR- and LR-types (see Table 2), especially for regions *E6 *(0.07 and 0.32, respectively), *E1 *(0.33 and 1.14, respectively), *L2 *(0.13 and 1.00, respectively), *L1 *(0.0 and 0.55, respectively), and LCR (0.0 and 0.46, respectively). In fact, among mucosal HR-HPV types, only HPV 33 contains a CGI in the *E6 *region; only HPV 45 contains a couple of CGIs in the *L2 *region, and none of the mucosal HR-HPVs contain CGIs in either the *L1 *or LCR regions.

Interestingly, when focusing only on regions where islands were identified (excluding any region with no CGI; see Table S3), the region possessing the highest CGI number is *L1 *(average 1.71 CGIs on 21 HPV types), followed by *L2 *(average 1.58 CGIs on 36 HPV types), *E1 *(average 1.26 CGIs on 61 HPV types) and LCR (average 1.20 CGIs on 25 HPV types). Oncogenes *E6 *and *E7 *as well as *E2 *and *E4 *ORFs share 1 CGI on 17, 45, 92 and 68 HPV types, respectively.

## Discussion

To our knowledge, this is the first systematic analysis of CGIs, as well as of CpG site distribution performed in 92 HPV types. This analysis provides the main structure underlying the methylation phenomenon, guiding the identification of interesting genomic regions for future epigenetic experimental studies.

As anticipated [23], we found significant differences between CpG observed and expected values among all sequenced HPV genomes. Nevertheless, contrary to the notorious underrepresentation of CpG sites among eukaryotic genomes where they account for only one third down to 5% of the expected values; viral genomes have an underrepresentation of only around 50% of the expected CpG values. According to the co-evolution hypothesis, it would be expected that both human and HPV genomes exhibit similar CpG underrepresentation values. The great differences found could be related to other relevant phenomena, such as the species-specific codon usage and the genomic base composition. In fact a relationship between codon use and base composition with C+G content in HPVs was previously found [34,35].

In this context, it seems necessary to mention that HPVs are small sized viral DNA genomes that do not evolve rapidly, in contrast to other viruses. It has been calculated that approximately 200,000 years are needed for 17 bp to change in these genomes [36].

In a biological context, a significant correlation between CpG observed and expected values could reflect global common functional constrictions shared by most HPV types. At the same time, the AsPCS inter-regional lack of correlations point to local functional constriction divergences, particularly in the *E6 *and LCR regions (Table 2). Nevertheless, more studies of different HPV types are necessary to confirm it.

Interestingly, *L2 *and *L1 *ORFs show a parallel CpG behavior. Both of them possess relatively low PCS arranged mostly in CGI. The *L2 *region is sixth place in average PCS value and fourth place in island number, and *L1 *is eighth place in average PCS value and sixth place in island number. It is known that in low grade cervical lesions associated to HPV 16, the viral genome is episomal, hypomethylated, and the *L1 *and *L2 *ORFs are expressed in order to generate viral capsids. In contrast, most high grade cervical lesions show viral DNA integrated, hypermethylated, and the *L1 *and *L2 *ORFs are not expressed. In vertebrates, methylation in body genes plays an important role in tissue- or cell-specific alternative promoter regulation, such that orphan CGIs are used for alternative promoter identification [37]. Even though this is a risky idea, it is tempting to speculate that viral capsid coding ORFs behave similarly and that when the cell hypermethylates them, it is possible that the *L1/L2 *region plays a role as an alternative promoter for viral genes and, when integrated, for some cellular genes.

Additionally, viral oncogenes also show a parallel CpG behavior, but in an opposite manner as the capsid coding genes. Both of them possess relatively high PCS values associated to few CGI. *E7 *occupies the second place according to average regional PCS values and the fifth place according to the average island number, while the *E6 *region occupies the fifth place according to regional PCS and the eighth place according to the average island number. This means CpG sites are widely distributed and suggest both regions could be sensitive to methylation, especially when the HPVs integrate in to the cell genome.

The *E4 *ORF seems to be a special case because of its multiple roles during the viral cycle. It is involved in diverse cell and viral functions, especially in diminishing keratinocyte integrity [38], cell cycle arrest at G2 [39], HPV DNA replication [40] and expression of late proteins [41], possibly through several proteolysis products from the E4 protein [42]. In fact, the *E4 *ORF is expressed throughout the viral cycle [2]. Its functions and continuous expression could impound hard functional restrictions to sequence mutations, as well as the need for a mechanism involved in avoiding methylation. The maintenance of CpG sites in CGIs could be a kind of compromise to meet sequence code restrictions with the necessary expression of the E4 protein.

In addition to the underrepresentation of CpG sites in HPV genomes, single nucleotide proportions point to T and A increased frequencies at the expense of G and C frequencies, reinforcing the C → T mutation hypothesis as a result of CpG deamination.

Nevertheless, the *E4 *region does not share the same behavior. Amazingly, even when A and G proportions are similar to those from the whole genome, C frequencies seem to be increased at the expense of Ts. This fact, added to the high number of CpG sites, mostly arranged into CGIs, leads to the question of whether there is any mechanism to avoid C → T mutations, resulting from deamination, that affects the HPV and specially the viral *E4 *region. In fact, there exist repair enzymes that remove deaminated bases; specifically, thymine DNA glycosylase (TDG) and methyl-CpG binding domain protein 4 (MBD4) which are able to remove either uracil or thymine from G·U and G·T miss-pairings where follow-on base excision repair enzymes restore a G·C pair [43,44].

When grouped by tropism, the cutaneous HPV group, shows the greatest CGI average size (446 bp), followed by the LR-HPV group with an intermediate CGI average size (363 bp) and the HR-HPV group shows the smallest average CGI size (272 bp). Additionally, even though CGIn from both cutaneous and mucosal HPV types share similar values (3 CGIs, average), it seems there is a trend toward smaller CGIn in HR- compared to LR- HPVs (1.6 and 3.93, respectively). These data raise questions about the possible role of CGIs on the interaction between virus and cells. At the same time, this suggests a possible differential susceptibility to methylation in function of either HPV tropism or associated risk. Nevertheless, the only experimental studies on methylomes have been carried out on HPV types 16 and 18, where methylation changes and high heterogeneity were observed along the viral infection phases [3-8]. In summary, those inter-group differences could be related to different viral infection epigenetic strategies.

Finally, it should be interesting to examine virus targeted genes in the human genome and see what their CGI and CpG features are. We hope that our analysis of CpG and CGI distribution among HPV genomes can provide elements for rational experimental approaches related to biological and evolutionary processes of these viruses and their hosts.

## Conclusions

First, we want to point out that this work is based solely on sequence feature analysis, so the results reported here may need experimental corroboration. In this study, we found that there are both significant differences and correlations between CpG observed and expected values among all sequenced HPV genomes, and that viral genomes have an underrepresentation of only around 50% compared to eukaryotic genomes where underrepresentation is from 30 down to 5%.

The regional analysis of CpG site distribution shows that correlations between complete genome PCS are significant, even though there is a lack of correlation between several genomic region pairs, especially those involving LCR and *E6*. Furthermore, we found that *L2 *and *L1 *ORFs exhibit an opposite behavior to oncogenes *E6 *and *E7*; the first pair possessing relatively low numbers of CpG sites which are associated to CGI while the oncogenes possess a relatively high number of CpG sites which are not associated to CGI. We would point out that *E4 *is the region possessing the highest content of CpGs in every HPV type where an *E4 *is identified.

From the CGI analysis, the *E2/E4 *is the only region with at least one CGI in every HPV, and the main difference between this region and the complete genome is that in the *E4 *region the frequency of Cs seems increased at the expense of Ts. The mucosal HR-HPVs possess the shortest CGI sizes, followed by the mucosal LR-HPVs and finally by the cutaneous viral subgroup. At the same time, the mucosal HR-HPVs show a trend toward lower CGI numbers, followed by the cutaneous viral subgroup and finally by the mucosal LR-HPVs.

Finally, we hope that our analysis of CpG and CGI distribution among HPV genomes can provide support for rational experimental approaches related to the biological and evolutionary processes of these viruses and their hosts.

## Methods

Our CpG analysis comprises two phases: the first one is the comparison of CpG site distribution among HPV types as a single group, and in the second phase we identify possible similarities or differences in CpG site distribution associated to the preferential tropism and/or the risk of cancer caused by the mucosal viral subgroups. The CpG distribution refers to CpG proportions (PCS) and either CGI sizes or numbers.

### Data Source

HPV DNA sequences were obtained online from GenBank (U.S. National Library of Medicine and National Institutes of Health; see additional file 1, Table S1). The main criterion for viral genome selection was that every nucleotide sequence had been previously identified as an HPV type by a taxonomic study. We selected all HPV sequences included in the classification schemes from De Villiers et al. [45], based on the L1 sequence, and from Diallo et al. [46], based on complete genome sequences.

Sequence from HPV type 16 includes the changes proposed by the Theoretical Biology and Biophysics Group in Los Alamos National Laboratory. All 92 HPV genomes included in the analysis were reedited so that all sequences begin at the first nucleotide from the *E6 *ORF. Exceptions to this are HPV types 14D and 71, because neither has a reported *E6 *ORF. Additionally, HPV types 14D and 3 lack reported *E7 *ORFs, HPV type 53 lacks a reported *E1 *ORF and 24 viral types lack reported *E4 *ORFs (HPV 1a, 3, 7, 8, 9, 10, 12, 14D, 15, 17, 19, 25, 26, 27, 30, 32, 34, 40, 45, 50, 52, 53, 56, and 71).

Because the absence of data may contribute to bias in the analysis, putative ORFs for each HPV type lacking reported ORFs were identified using the online tool "ORF finder" [47]. The main criteria for putative ORF selection was the size of the identified coding regions and the existence of only one candidate ORF in each considered region. We took into account single fragments of 200 bp or greater, and then we calculated the PCS for these putative ORFs. As can be seen in Additional File 4, Table S4, we found putative single *E*1 and *E*7 ORFs for HPV types 53 and 3, respectively. In the case of HPV 14D, we found single *E*6 and *E*7 ORFs, however these were found to overlap by more than 100 bp. For HPV types without a reported *E*4 ORF, we identified a single putative ORF in only three viral types: HPV 25, 26 and 32. The other 21 HPV types had more than one identified ORF: we found 11 HPV types with 2, 8 HPV types with 3, 1 HPV type with 4 and 1 HPV type with 5 putative *E*4 ORFs.

The PCS for the putative ORFs did not notably change the regional PCS averages nor the analysis of the results (See additional file 5, Table S5).

Finally, in order to avoid bias in the results, we included one sequence from each HPV type and avoided any variant type sequences.

### Input Data

The input data comprehended the entire genome, each ORF and LCR, including start and end sites, for every HPV type.

We analyzed the entire genomes of 92 viral sequences, as well as dividing them into 8 regions (*E1, E2, E4, E6, E7, L1*, and *L2 *ORFs, and LCR). Because of the scarce amount of data, we did not include three reported ORFs into the analysis: the *E8 *ORF has been found only in HPV 1; the *E5 *ORF is present in only 23 out of 92 HPV types (5, 6a, 11, 13, 16, 18, 31, 33, 35, 39, 41, 42, 51, 58, 59, 67, 68, 69, 70, 74, 82 and 85), and the *E5*B ORF is only reported for two HPV types (11 and 31).

### Output Data

**Figure 3:**
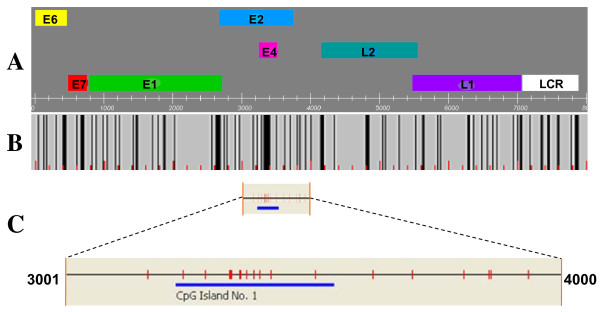
**CpG distribution in the genome of HPV type 16**. A. Viral gene map; B. Location of CpG sites, and C. Single CpG island identified.

In order to identify the CpG sites we used PISMA, a computational tool we designed, which is available upon request to SCG and/or RAS. PISMA allows the location and identification of motifs ranging between 2 to 10 bases, in up to 10 kb DNA sequences. In addition, this tool will count the number of motifs in defined regions. We have identified the individual CpG sites per region from all HPV sequences, and based on those data from PISMA, we calculated the PCS as the number of CpG sites divided by total number of nucleotides in the sequence. Identification of putative CpG islands/clusters was carried out using CpG island Explorer 2.0, publically available in http://www.uscnorris.com/cpgislands2/cpg.aspx[28]; parameters used in CpG island identification were those from Gardiner-Garden and Frommer [26]: CpG 50% or more, ObsCpG/ExpCpG ratio 0.60 or higher, at least 200 bp size and 100 bp gap. In Figure 3 results obtained for HPV 16 are shown.

PCS Arc-sin transformations were calculated in order to properly use parametric statistical procedures that are not suitable for proportion data because of the binomial nature of the biological process (CpG occurrence). In summary, the distribution of proportions is often skewed, and the arcsine transformation often makes that distribution more normal.

Finally, we calculated the CpG expected values for each viral genome in order to compare them with the CpG observed values.

### Data Analysis

Possible relationships between data results were identified using rho Spearman Rank Correlation and Chi square tests, as well as Pearson Product Moment Correlation and two sided t tests. Calculations were carried out using *ad hoc *Excel macros and corroboration of calculations, using free online statistics software [48,49].

### Revision Procedure

In order to check for possible mistakes in the calculations, we reviewed the whole procedure by randomly selecting three groups of thirty HPV types each. One group was used to repeat the input data procedures; a second group was used to repeat the output data procedures, and the third group was used to repeat statistical and p value calculations.

## List of abbreviations

HPV: human papillomavirus; LR-HPV: low-risk HPV types; HR-HPV: high-risk HPV types; ORF: open reading frame; PCS: proportion of CpG sites; AsPCS: Arc-sin transformed proportion of CpG sites; m5CpG: methylated CpG site; CGI: CpG island; CGIn: CpG island number; E(number): viral early protein; L(number): viral late protein; *E*(number): viral early ORF; *L*(number): viral late ORF; LCR: long control region; SRPK1: serine-arginine-rich protein kinase 1.

## Authors' contributions

SCG initiated the study, performed the analysis and interpretation, and wrote the manuscript. MMS, VMG and RM obtained the data and revised the manuscript. RAS, GTDC and MAH developed the computational tool and participated in obtaining the data, and AGC provided initial impetus to perform the analysis and participated in writing and reviewing the manuscript. All authors read and approved the final manuscript.

## Authors' information

SCG is an associate researcher and RAS is a professor at the National University of Mexico; AGC is a researcher at the National University of Mexico and at the National Cancer Institute; VMG is a professor at the Metropolitan University; RM is an associate researcher at the National Cancer Institute; MMS is post doctoral fellow at the AGC lab; GTDC and MAH are students guided by RAS.

## Supplementary Material

Additional file 1 **Table S1. CpG observed and expected values for each HPV type**. The table contains the GenBank accession number, the CpG observed and expected numbers, and the CpG O/E ratio, for all 92 HPV types included in the analysis. Click here for file

Additional file 2 **Table S2. Proportion of CpG sites among different genomic regions**. The table contains the proportion of CpGs in the *E6, E7, E1, E2, E4, L2 *and *L1 *ORFs, and the LCR, the entire genome and all ORFs together, for every viral type. Click here for file

Additional file 3 **Table S3. CpG islands per region from each HPV type**. The table contains the CpG island number in the *E6, E7, E1, E2, E4, L2 *and *L1 *ORFs, and the LCR and of entire genome, the CGI average size and the tropism for every viral type. Click here for file

Additional file 4 **Table S4. Putative ORFs from HPV types lacking reported ORFs**. The table contains every possible coding fragment 200 bp or larger for each HPV type lacking reported ORFs; the starting and ending sites, and the corresponding PCS values. Click here for file

Additional file 5 **Table S5. p values for correlations between regional AsPCS when putative ORFs are included**. The table contains p values for correlations between regional AsPCS values when AsPCS from putative ORFs are included. Click here for file
